# Safety and Efficacy of Combined Low-Dose Lithium and Low-Dose Aspirin: A Pharmacological and Behavioral Proof-of-Concept Study in Rats

**DOI:** 10.3390/pharmaceutics13111827

**Published:** 2021-11-01

**Authors:** Rachel Shvartsur, Galila Agam, Alla Shnaider, Sarit Uzzan, Ahmad Nassar, Adi Jabarin, Naim Abu-Freha, Karen Meir, Abed N. Azab

**Affiliations:** 1 Department of Nursing, School for Community Health Professions, Faculty of Health Sciences, Ben-Gurion University of the Negev, Beer-Sheva 8410501, Israel; shvartsurr@gmail.com; 2 Department of Clinical Biochemistry and Pharmacology, Faculty of Health Sciences, Ben-Gurion University of the Negev, Beer-Sheva 8410501, Israel; galila@bgu.ac.il (G.A.); sarituzzan@gmail.com (S.U.); nassarah@post.bgu.ac.il (A.N.); adi.jbaren@gmail.com (A.J.); 3 Department of Nephrology, Soroka University Medical Center, Beer-Sheva 84101, Israel; ellasc@bgu.ac.il; 4 The Institute of Gastroenterology and Hepatology, Soroka University Medical Center, Beer-Sheva 84101, Israel; abufreha@yahoo.de; 5 Department of Pathology, Hadassah Medical Center and Faculty of Medicine, The Hebrew University, Jerusalem 91120, Israel; karenm@hadassah.org.il

**Keywords:** aspirin, bipolar disorder, inflammation, lithium, nephrotoxicity

## Abstract

Despite established efficacy in bipolar disorder patients, lithium (Li) therapy has serious side effects, particularly chronic kidney disease. We examined the safety and behavioral effects of combined chronic low-dose aspirin plus low-dose Li in rats to explore the toxicity and therapeutic potential of this treatment. Rats were fed regular or Li-containing food (0.1% [low-dose, LLD-Li] or 0.2% [standard-dose, STD-Li]) for six weeks. Low-dose aspirin (1 mg/kg) was administered alone or together with Li. Renal function and gastric mucosal integrity were assessed. The effects of the combination treatment were evaluated in depression-like and anxiety-like behavioral models. Co-treatment with aspirin did not alter plasma Li levels. Chronic STD-Li treatment resulted in significant polyuria and polydipsia, elevated blood levels of creatinine and cystatin C, and increased levels of kidney nephrin and podocin—all suggestive of impaired renal function. Aspirin co-treatment significantly damped STD-Li-induced impairments in kidney parameters. There were no gastric ulcers or blood loss in any treatment group. Combined aspirin and LLD-Li resulted in a significant increase in sucrose consumption, and in the time spent in the open arms of an elevated plus-maze compared with the LLD-Li only group, suggestive of antidepressant-like and anxiolytic-like effects, respectively. Thus, we demonstrate that low-dose aspirin mitigated the typical renal side effects of STD-Li dose and enhanced the beneficial behavioral effects of LLD-Li therapy without aggravating its toxicity.

## 1. Introduction

Bipolar disorder (BD) is a chronic psychiatric disorder characterized by repeated manic, hypomanic, and depressive episodes [1,2,3,4,5]. It affects 1–2% of the general population [1,2,6] and is recognized as one of the leading disability causes worldwide, associated with significant impairment in work, family, and social life, beyond the acute phases of the illness [2,7]. BD patients suffer from an increased incidence of comorbidities which further aggravate their physical and functional status [4,8,9]. For example, bipolar patients have significantly higher rates of myocardial infarction [10,11], stroke [10], atherosclerosis [12], and hypertension [13] than the general population [9]. Cardiovascular disease is the leading cause of death in BD [14], with patients having a two-fold increased risk of mortality from coronary artery disease as compared to matched control subjects [8]. Moreover, studies indicate that amongst all psychiatric disorders, BD is associated with the highest rate of suicide death [15,16].

Lithium (Li) salts are useful for the maintenance treatment of BD [3,5]. Li has established efficacy preventing recurrence of manic as well as depressive episodes [3,5,17,18,19,20,21] and an ability to reduce suicidal death among bipolar patients [15,22,23,24]. Nonetheless, despite its proven therapeutic efficacy, the use of Li has several disadvantages [19,22,25,26,27,28,29]: (1) The drug has a narrow therapeutic window requiring regular therapeutic drug monitoring. The serum concentration range for Li maintenance treatment regarded as effective, while retaining a reduced toxicity risk, is between 0.6 and 1.2 mEq/L; (2) the use of Li for the treatment of acute manic and depressive episodes in BD patients is questionable given the lag between commencement of Li intake and emergence of its therapeutic effects; (3) a high percentage of patients do not respond to treatment and suffer recurrent manic and depressive episodes; and, (4) Li treatment is associated with a wide range of adverse effects.

A particular concern has been the effect of Li on renal function [28,30,31,32,33]. The most common renal side effect of Li is nephrogenic diabetes insipidus (NDI) characterized by polyuria, polydipsia, and a decrease in urinary concentrating ability and unresponsiveness to vasopressin [31]. Factors that contribute to the development of NDI are elevated blood Li levels, long treatment duration, and high incidence of Li intoxication episodes [31]. Moreover, an unignorable body of data suggests that long-term use of Li increases the risk of chronic kidney disease (CKD) [30,31,32,33,34,35,36,37,38,39,40]. For example, Rej et al. [32] found that Li is independently associated with an almost two-fold increase in CKD risk in elderly adults [32]. Nevertheless, it could still be argued that inconsistencies exist across the literature, as some studies did not find a causative association between Li treatment and CKD [30,41]. Importantly, it has been suggested that administration of low to medium Li doses may reduce the incidence of CKD and enhance adherence to treatment [31,42,43,44].

Ample data points to the involvement of inflammation in the pathophysiology of BD [45,46,47,48,49,50,51,52]. Epidemiologic studies identified elevated rates of BD among patients with inflammation-associated comorbidities, including inflammatory bowel disease [53,54], rheumatoid arthritis [53,54,55], systemic lupus erythematosus [56], psoriasis [53,57], multiple sclerosis [53,58], obesity [54], and type 2 diabetes mellitus [54,59]. Examining the inflammatory profile of bipolar patients revealed elevated blood levels of inflammatory mediators, especially during acute mood episodes [47,48,51,52]. Moreover, levels of inflammatory markers were found higher in postmortem brain [60,61] and cerebrospinal fluid of BD patients as compared to matched control subjects [62,63,64,65]. Consistently, evidence indicates that psychotropic drugs exert a variety of anti-inflammatory effects [17,49,66,67,68,69,70,71,72,73,74] which may contribute to their therapeutic efficacy. Accordingly, anti-inflammatory drugs such as corticosteroids and nonsteroidal anti-inflammatory drugs (NSAIDs) have been shown to reduce the severity of symptoms among psychiatric patients [45,47,48,75,76,77,78,79,80,81,82,83].

Aspirin (acetylsalicylic acid, ASA) is an NSAID that exerts dose-dependent [25,84] antiplatelet, analgesic, antipyretic and anti-inflammatory effects. High aspirin doses (>325 mg/day in adult humans) inhibit cyclooxygenase COX-1 and COX-2, resulting in antipyretic and mild to moderate analgesic effects. On the other hand, low aspirin doses (75–150 mg/day) preferentially and irreversibly inhibit COX-1, resulting in inhibition of platelets aggregation and clot formation [25,84]. In patients with stable cardiovascular disease, low-dose aspirin therapy reduces the incidence of adverse cardiovascular events and all-cause mortality [85,86,87] but, concomitantly, it increases the risk for bleeding complications, including gastrointestinal bleeding and intracranial hemorrhage [85,88]. Nevertheless, discontinuation of aspirin in patients with coronary artery disease may increase the risk for major adverse cardiovascular events [85,89]. Therefore, the American College of Gastroenterology supports the use of aspirin when the beneficial cardiovascular effects outweigh the gastrointestinal risks [90,91].

In mood disorders (including BD), reports support a possible beneficial therapeutic effect of low-dose aspirin [92,93,94,95]. A Danish population-based study [95] found that continued use of low-dose aspirin was associated with significantly decreased incidence and hazard risk of BD. Savitz et al. [94] reported a beneficial effect of low-dose aspirin as a treatment for bipolar depression. Others reported beneficial effects of aspirin in major (unipolar) depression [92,93].

In order to gain the exceptional therapeutic benefits of Li, it is crucial to search for new strategies to cope with its nephrotoxicity. As mentioned, a large body of data indicates that the use of low to medium Li doses (below those regarded as therapeutically relevant) may reduce the incidence of CKD and increase adherence to treatment [31,42,43,44]. Circumstantial evidence suggested that co-administration of low-dose aspirin with Li may enhance the therapeutic efficacy of Li [96]. In the present study, we investigated the possible beneficial effect of low-dose aspirin as an add-on to low-dose Li treatment. Towards this goal, we first probed the safety of chronic administration of combined low-dose aspirin and Li in rats. In particular, determinants of renal function were measured to establish whether or not the combination of low vs. standard Li dose plus low-dose aspirin aggravates the renal side effects of Li. We then assessed whether co-administration of low-dose aspirin with low-dose Li preserves the efficacy of therapeutically relevant Li doses while reducing toxicity.

## 2. Materials and Methods

### 2.1. Animals

Male Sprague-Dawley rats weighing 220–250 g at the beginning of the experiments were used in the study. The inclusion criteria of only male rats emanated from two main considerations: (i) The ethical principle to use the minimal possible number (appropriate for statistical analysis) of animals in a “proof-of-concept” study; and, (ii) the known gender-related differences in lithium’s kinetics in male vs. female rats [97] which would have complicated the statistical analysis of the results. Rats were maintained under controlled environmental conditions (ambient temperature 22 ± 1 °C, relative humidity 45–55%, photoperiod cycle 12 h light: 12 h dark), with food and water ad libitum unless otherwise indicated. Upon arrival to the animal facility, rats were allowed to adapt to the new environment for one week prior to the initiation of treatment and behavioral studies. All experiments complied with the ARRIVE guidelines and were carried out in accordance with the guidelines of the Committee for the Use and Care of Laboratory Animals in Ben-Gurion University of the Negev, Israel (Approval # IL-45-08-2017).

### 2.2. Chronic Treatment with Li and Aspirin

Rats were fed 0.1% or 0.2% (*w*/*w*) Li-containing diet (in regular powdered rodent chow) for 42 days. Control rats were fed an identical diet, but without added Li (regular food). We modified previous Li treatment protocols [74,98] and established a protocol through which we obtained two categories of Li-treated rats according to their plasma Li concentrations. The categories were: (1) standard-dose Li (STD-Li, 0.2% Li in food) resulting in therapeutically-relevant plasma Li concentrations between 0.6–1.2 mEq/liter [22,26], and, (2) low-low-dose Li (LLD-Li, 0.1% Li in food) resulting in plasma Li concentrations between 0.2–0.4 mEq/liter. Low-dose aspirin (1 mg/kg, intraperitoneally [ip] [99]), alone or together with Li, was administered for 42 days. This duration in rats parallels nearly 4–10 years in humans [100,101]. In addition to food and tap water available ad libitum, all groups had free access to an additional bottle of 0.9% NaCl solution to prevent electrolyte imbalance and mitigate the risk for Li intoxication due to decreased renal clearance. This practice is routinely done in chronic Li treatment studies in rodents [98,102,103,104] in order to prevent polyuria-induced lithium intoxication due to hyponatremia (which induces over-reabsorption of lithium) [105,106,107]. It is usually not associated with changes in plasma sodium levels [108] due to regulatory-compensatory biological mechanisms which maintain sodium levels at normal levels. Consistently, as presented in Section 3.3, plasma sodium levels did not differ significantly between the groups.

### 2.3. Toxicity Assessment

Toxicity was evaluated at two levels—renal function and gastric mucosal integrity and bleeding. These determinants were assessed because chronic treatment with Li and aspirin is associated with impairment of renal function and disruption of gastric mucosal integrity (and ulceration), respectively. In two out of four independent *“Toxicity Experiments*”, on treatment days 21 and 42, half of the animals in each group were euthanized by decapitation. Then, stomachs and kidneys were rapidly excised, kidneys were weighed, stomachs macroscopically inspected and immediately frozen at −80 °C for further analysis. One kidney was frozen at −80 °C for later use in enzyme-linked immunosorbent assay (ELISA) experiments and the second was preserved in formaldehyde for histopathological examination. Determinants measured to evaluate renal function included water consumption, urinary output, histopathological examination to detect the presence of tubular cysts and tubulointerstitial damage, blood levels of creatinine, urea, and cystatin C, and levels of the proteins nephrin and podocin in renal tissue. Cystatin C is a biomarker of early kidney injury which helps detect early impairment in glomerular filtration [109,110]. Nephrin and podocin are proteins that are crucial for podocyte function (podocytes form the glomerular epithelium which plays a pivotal role in the blood-to-urine filtration barrier) [111]. Alterations in activity/expression of nephrin and podocin lead to impairment in glomerular function [112,113].

#### 2.3.1. Assessment of Water Consumption

Bottles of water/saline were weighed before and after test sessions. The net amount of consumed liquid spanning over 24 h was divided by the rats’ weight.

#### 2.3.2. Determination of Urinary Output

At time zero (T 0 h), rats were placed in pre-weighed cages containing known amounts of dry sawdust. After 24 h (T 24 h) the wet sawdust containing the urine was cleaned of rats’ feces and cages were re-weighed. Urinary output was calculated as [T 24 h cage weight—T 0 h cage weight]/rats’ body weight. Food was placed in specially designated heavyweight bowls to avoid rats turning them upside down and avoid food dropping to the sawdust. The urine volumes that we detected in the control and Li-treated rats using the above-described method were similar to those reported in previous studies which used a different methodology for measuring urine volume in control and chronically Li-treated rats [114,115]. Of note, conduction conditions were meticulously designed to obviate the phenomenon of urine evaporation (data not shown).

#### 2.3.3. Determination of Plasma Li, Creatinine, Urea and Cystatin C Levels

Blood was withdrawn by tail vein puncture (1.0 mL into heparin-containing 1.5 mL tubes, on day 0) or after decapitation (6–8 mL into heparin-containing 15 mL tubes, on day 21 or 42), centrifuged at 3500× *g*, 4 °C for 10 min. Plasma was separated by aspiration using a narrow-necked pipette, inserted into heparin-containing tubes, and kept at −80 °C until further assessment. Li, creatinine, and urea levels were detected in the Biochemistry lab of Soroka University Medical Center (SUMC), Beer-Sheva, Israel. Li levels were measured using an Ion-Selective Electrode (ISE) electrolyte analyzer (Cobas Integra 400, Roche Diagnostics, Rotkreuz, Switzerland); creatinine and urea were measured using an Olympus Beckman Coulter AU5800 apparatus (Brea, CA, USA). Cystatin C levels were determined by a specific ELISA kit (Boster Bio, Pleasanton, CA, USA).

#### 2.3.4. Determination of Nephrin and Podocin Levels

One half (longitudinal section) of the right kidney (arbitrarily chosen) of each rat was weighed and homogenized on ice (by a Polytron PT 1200 E Hand Disperser, Kinematica, Malters, Switzerland) in a buffer containing a protease/phosphatase inhibitor cocktail (1:10 *w*/*w* Phosphatase Inhibitor Cocktail x 100 in ddH_2_O, APExBIO; Protease Inhibitor Cocktail X 100 in DMSO, APExBIO). The homogenate was centrifuged at 12,750× *g*, 4 °C for 10 min, and then supernatant and pellet were separated and immediately frozen at −80 °C. Nephrin and podocin levels were determined in the supernatant fraction using specific ELISA kits (A&E Scientific, Mark, Belgium).

#### 2.3.5. Determination of Renal Tubulointerstitial Damage

Histopathological examination to assess the existence of tubular cysts and tubulointerstitial damage was performed under the guidance of a nephrologist (A.S.) as follows: the left kidney of each rat was fixed in buffered formalin (paraformaldehyde solution 4% in phosphate-buffered saline [PBS], Santa Cruz Biotechnology, Dallas, TX, USA) and embedded in paraffin. The kidney was then longitudinally sectioned in half, 2 μm sections were cut, stained with hematoxylin and eosin and with Periodic acid–Schiff (PAS), examined in an Olympus Bx41 microscope (Tokyo, Japan), and photographed with a digital camera (Olympus DP72, Tokyo, Japan). The assessment was performed independently by a nephrologist (A.S.) and by a pathologist (K.M.) who were blind to the treatment.

#### 2.3.6. Assessment of Gastric Mucosal Damage and Bleeding

The macroscopic presence of gastric erosions/ulcers was examined by a gastroenterologist (N.A.-F.) immediately after euthanizing the rats. Thereafter, stomachs were frozen at −80 °C for further analysis. Upon defrosting, 100 mg of the gastric tissue (from the same zone) were homogenized as described above for the kidney (2.3.4) and mucosal PGE2 levels were determined using a specific ELISA kit (R&D Systems, Minneapolis, Minnesota). Possible blood loss (bleeding) was assessed by measuring blood hemoglobin levels and red blood cell count in the Hematology lab in SUMC, and plasma thromboxane A2 (TXA2) levels were determined using a dedicated ELISA kit (MyBioSource, San Diego, CA, USA).

### 2.4. Behavioral Studies

All behavioral studies were conducted during the light phase, at least 2 h after the administration of aspirin. The following behavioral tests were performed in order to determine the therapeutic potential of the combined treatment.

#### 2.4.1. Open Field Test (OFT)

OFT was used to assess the spontaneous activity of animals [116]. Rats were placed for 10 min in the corner of an open field arena made of a black lusterless perspex box (60 cm [W] × 80 cm [L] × 60 cm [H]). Sessions were videotaped by a camera placed approximately 1 m above the center of the arena and subsequently assessed using a video-tracking system (Ethovision, Noldus, Wageningen, Netherlands). A 5% ethanol in water was used to clean the apparatus prior to the introduction of each animal. The parameters analyzed were total distance traveled and time spent in the central zone and the peripheral zone of the arena.

#### 2.4.2. Sucrose Consumption Test (SCT)

SCT was used to assess anhedonia, a behavioral facet of depression. The test was conducted as previously described [116]. Briefly, animals were exposed once to 1% sucrose (Sigma, St. Lewis, MO, USA) solution to customize them to the procedure before conducting the actual experiment. During test sessions, rats were supplied with a bottle of sucrose solution in addition to the regularly supplied water bottle for 24 h. Food was freely available. Sucrose consumption was calculated as the decrement between the weight of the sucrose bottle at the beginning of the experiment and 24 h thereafter divided by the bodyweight of all rats in the given cage. The test was conducted under similar conditions at baseline, and at 3 and 5 weeks after the commencement of treatment.

#### 2.4.3. Elevated Plus-Maze Test (EPMT)

EPMT was used to measure anxiety-like behavior (associated with depression) and risk-taking behavior (associated with mania) [117]. Naturally, rodents hide in dark places during light time, to avoid the possibility of being seen by enemies (predators). Thus, the choice to enter the open arms of the plus-maze represents a normal, non-anxious behavior when the proportion of time spent in these arms is similar or not significantly higher than in control animals. Alternatively, going to the open arms may also represent a risk-taking behavior, because it suggests that the animal is not afraid to take the risk of being seen by enemies [117]. Rats were placed for 5 min in an elevated maze consisting of two open arms (50 cm × 10 cm) and two walled arms with an open roof (50 cm × 10 cm × 40 cm), elevated to a height of 50 cm and arranged such that the two open arms are opposite to each other. Rats were placed in the center of the maze, facing one of the open arms. Sessions were videotaped by a camera placed 2 m above the center of the maze and subsequently assessed using a video-tracking system (Ethovision, ibid). The following parameters were evaluated: number of entries and time spent in the open and walled arms. An arm entry was defined as the entry of all four limbs into the arm.

### 2.5. Statistical Analysis and Presentation of the Results

All quantitative results are expressed as mean ± SEM. Differences among the means of multiple parameters were analyzed by two-way analysis of variance (ANOVA) and Student’s *t*-test. After ANOVA, Fisher’s post hoc test was performed to compare each of the groups to the others. Differences among non-parametric variables such as gastric mucosal integrity were analyzed using the chi-square test. Values of *p* < 0.05 were considered statistically significant. We performed four independent *Toxicity Experiments* and two independent *Behavioral Experiments*. The original number of rats in the treatment groups was as follows: Control = 9, LLD-Li = 12, STD-Li = 12, Aspirin = 9, LLD-Li + Aspirin = 12, STD-Li + Aspirin = 12. Animals were excluded from the analysis only due to ethical considerations (e.g., if they presented unusual or sickness behavior, or significant changes in physiological parameters) or technical limitations (e.g., insufficient amount of blood for hematological analysis at time “zero”). Thus, the number of animals per group was not identical for all the tested parameters; the precise sample sizes are presented accordingly in each figure.

## 3. Results

### 3.1. Study Groups

A typical experiment included the following six groups: (1) Control—fed regular food and administered vehicle; (2) LLD-Li and vehicle; (3) STD-Li and vehicle; (4) Aspirin (only); (5) Aspirin + LLD-Li; (6) Aspirin + STD-Li.

### 3.2. Plasma Li Levels following the Various Regimens of Chronic Aspirin + Li Co-Treatment

**Figure 1:**
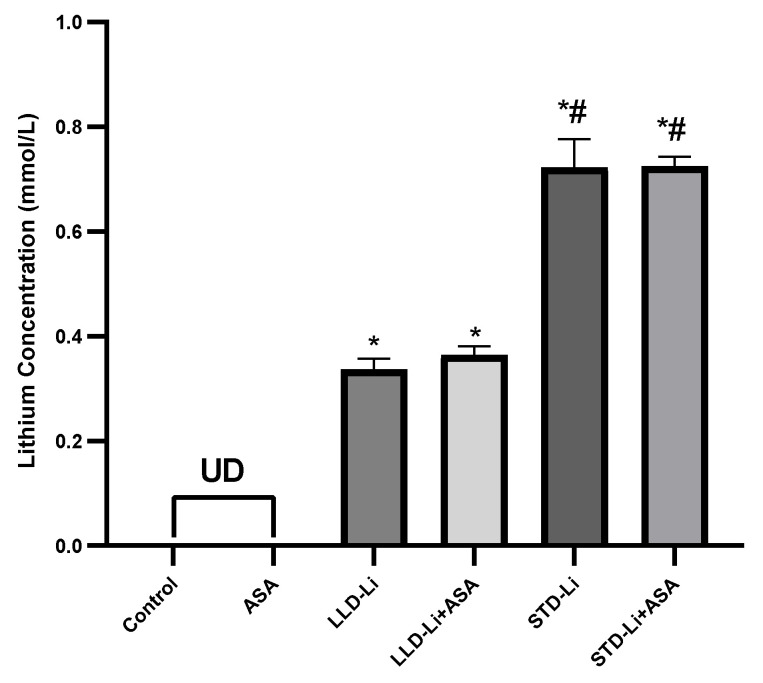
Li blood levels in aspirin + Li-treated rats. Rats were fed regular food (control) or Li-containing food [0.1% (LLD-Li), 0.2% (STD-Li)] for 42 days. Low-dose ASA (1 mg/kg, ip) was administered alone or together with Li. Blood was collected on days 0 and 42 of treatment, plasma separated, and Li levels determined as described in Materials and Methods. Li levels were undetectable in all groups on Day 0 and thus are not presented in the Figure. Results are the means ± SEM of a single representative experiment out of two demonstrating a similar pattern with 9–12 rats per group in the depicted experiment. * *p* < 0.0001 vs. Control, # *p* < 0.0001 vs. LLD Li. Two-way ANOVA: ASA effect: F_1,59_ = 0.1849, *p* = 0.6687; Li effect: F_2,59_ = 328.6, *p* < 0.0001; aspirin x Li interaction: F_2,59_ = 0.1608, *p* = 0.8519. Post hoc Fisher’s LSD: Control vs. LLD-Li or STD-Li—*p* < 0.0001, LLD-Li vs. STD-Li—*p* < 0.0001, LLD-Li vs. LLD-Li + ASA—*p* = 0.4687, STD-Li vs. STD-Li + ASA—*p* = 0.9644. Abbreviations: ASA—acetylsalicylic acid, LLD—low-low dose, Li—lithium, STD—standard dose, UD—undetectable.

Following 42 days of treatment, plasma Li levels in the LLD-Li group were 0.34 ± 0.07 mEq/L, and 0.72 ± 0.18 mEq/L in the STD-Li group (Figure 1). Co-treatment of Li with low-dose aspirin did not alter plasma Li levels in all regimens (two-way ANOVA, aspirin effect *p* = 0.669), indicating that the combined treatment is safe and does not increase the risk for Li intoxication.

### 3.3. Addition of Aspirin to Li Does Not Affect Water Consumption and Urinary Output

**Figure 2:**
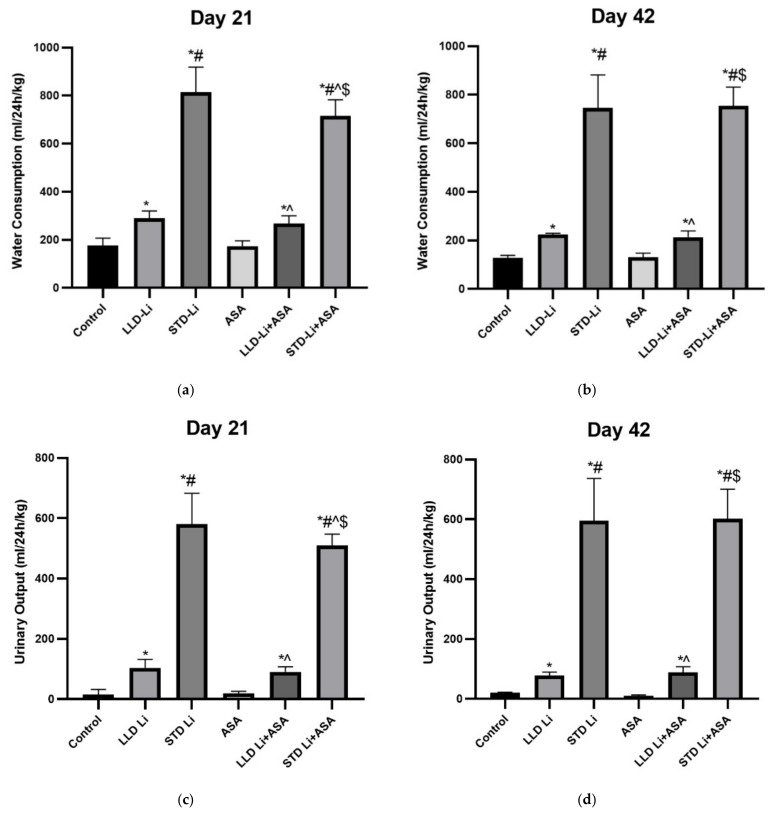
Water consumption and urinary output in aspirin + Li-treated rats. Rats were fed regular food (control) or lithium-containing food [0.1% (LLD) or 0.2% (STD)] for 42 days. Low-dose aspirin (1 mg/kg, ip) was administered alone or together with Li. At indicated days, 24 h water consumption (**a**,**b**) and urinary output (**c**,**d**) were measured as described in Materials and Methods. Results are means ± SEM of a single representative experiment out of two demonstrating a similar pattern with 9–12 (day 21) or 6 (day 42) rats per group in the depicted experiment. (**a**) Water consumption day 21, two-way ANOVA: ASA effect, F_1,59_ = 7.938, *p* = 0.007; Li effect, F_2,59_ = 635.4, *p* < 0.0001; aspirin x Li interaction, F_2,59_ = 4.091, *p* = 0.021. Post hoc Fisher’s LSD test: Control vs. LLD-Li; Control vs. STD-Li, *p* < 0.0001; LLD-Li vs. STD-Li, *p* < 0.0001; LLD-Li vs. LLD-Li + ASA, *p* = 0.34; STD-Li vs. STD-Li + ASA, *p* = 0.0001. (**b**) Water consumption day 42, two-way ANOVA: ASA effect, F_1,30_ = 2.689 × 10^−5^, *p* = 0.9959; Li effect, F_2,30_ = 320.5, *p* < 0.0001; aspirin X Li interaction: F2,30 = 0.069, *p* = 0.9328. Post hoc Fisher’s LSD test: Control vs. LLD-Li, *p* = 0.015; Control vs. STD-Li, *p* < 0.0001; LLD-Li vs. STD-Li, *p* < 0.0001; LLD-Li vs. LLD-Li + ASA, *p* = 0.77; STD-Li vs. STD-Li + ASA, *p* = 0.83. (**c**) Urinary output day 21, two-way ANOVA: ASA effect, F_1,59_ = 4.528, *p* = 0.037; Li effect: F_2,59_ = 729.5, *p* <0.0001; aspirin X Li interaction, F_2,59_ = 3.384, *p* = 0.04. Post hoc Fisher’s LSD test: Control vs. LLD-Li, *p* = 0.0002; Control vs. STD-Li, *p* < 0.0001; LLD-Li vs. STD-Li, *p* < 0.0001; LLD-Li vs. LLD-Li + ASA, *p* = 0.523; STD-Li vs. STD-Li + ASA, *p* = 0.0009. (**d**) Urinary output day 42, two-way ANOVA: ASA effect, F_1,30_ = 0.0044, *p* = 0.9475; Li effect, F_2,30_ = 241.2, *p* < 0.0001; aspirin X Li interaction, F_2,30_ = 0.07569, *p* = 0.9273. Post hoc Fisher’s LSD test: Control vs. LLD-Li, *p* < 0.0001; Control vs. STD-Li, *p* < 0.0001; LLD-Li vs. STD-Li, *p* < 0.0001; LLD-Li vs. LLD-Li + ASA, *p* = 0.80; STD-Li vs. STD-Li + ASA, *p* = 0.895. Asterisks and symbols denote the following: *—*p* < 0.05 vs. control; #—*p* < 0.05 vs. LLD-Li; ^—*p* < 0.05 vs. STD-Li; $—*p* < 0.05 vs. LLD-Li + ASA. Abbreviations: ASA—acetylsalicylic acid, LLD—low-low dose, Li—lithium, STD—standard dose.

Li was administered alone or together with aspirin for 42 days and water consumption and urinary output were determined on days 21 and 42. At baseline, water consumption and urinary output did not differ between the groups (data not shown). As expected, water consumption was significantly higher in Li groups at 21 and 42 days of treatment, as compared to control (two-way ANOVA, lithium effect *p* < 0.0001) (Figure 2a,b, respectively). Of note, water consumption was significantly higher in the STD-Li group as compared to the LLD-Li group (*p* < 0.0001). Importantly, co-treatment with aspirin did not aggravate the increase in water consumption; on the contrary, the addition of aspirin significantly reduced water consumption of the STD-Li group at 21 days of treatment (*p* = 0.0001) (Figure 2a). Similar results were obtained for urinary output (Figure 2c,d). Namely, the addition of aspirin significantly decreased the elevation in the STD-Li group at 21 days of treatment (*p* = 0.0009). These findings reinforce our hypothesis that add-on therapy of low-dose aspirin to Li is safe and does not exacerbate the renal side effects of Li. Furthermore, taking into account the prominent polyuria that occurs in Li-treated rats, we examined the effect of Li on plasma sodium levels to exclude a condition of Li-induced hypernatremia. Plasma sodium levels did not differ significantly between control and Li-treated rats after six weeks of treatment (Control = 139.9 ± 6.8, LLD-Li = 139.5 ± 6.2, STD-Li = 137.6 ± 6.6, ANOVA—*p* = 0.5019). Aspirin treatment also did not alter plasma sodium levels.

### 3.4. Effects of Chronic Aspirin plus Li Treatment on Kidney Parameters

#### 3.4.1. Creatinine

**Figure 3:**
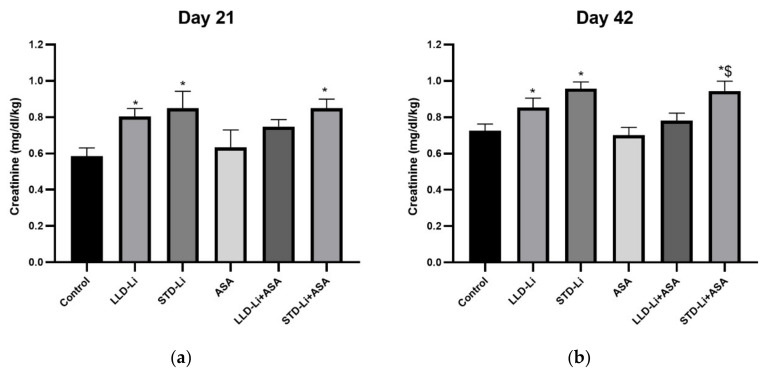
Plasma creatinine levels in aspirin + Li-treated rats. Rats were fed regular food (control) or Li-containing food (LLD-Li or STD-Li) for 42 days. Low-dose ASA (1 mg/kg, ip) was administered alone or as add-on to Li. On days 21 (**a**) and 42 (**b**) blood was collected, plasma separated, and creatinine levels determined as described in Materials and Methods. Presented are creatinine levels adjusted to rat’s body weight. The figure summarizes the combined results of two independent experiments demonstrating a similar pattern. Results are the means ± SEM of 9–12 rats per group. (**a**) Two-way ANOVA: ASA effect, F_1,64_ = 0.6556, *p* = 0.6874; Li effect, F_2,64_ = 10, *p* = 0.0002; aspirin x Li interaction: F_2,64_ = 0.3770, *p* = 0.6874. Post hoc Fisher’s LSD test: Control vs. LLD-Li, *p* = 0.0274; Control vs. STD-Li, *p* = 0.0005; Control vs. STD-Li + ASA, *p* = 0.0064. (**b**) Two-way ANOVA: aspirin effect, F_1,112_ = 1.031, *p* = 0.312; Li effect, F_2,112_ = 14.79, *p* < 0.0001; aspirin X Li interaction: F_2,112_ = 0.2559, *p* = 0.775. Post hoc Fisher’s LSD test: Control vs. LLD-Li, *p* = 0.045; Control vs. STD-Li, *p* = 0.0003; Control vs. STD-Li + ASA, *p* = 0.0006; LLD-Li + ASA vs. STD-Li + ASA, *p* = 0.0095. Asterisks and symbols denote the following: *—*p* < 0.05 vs. Control, $—*p* < 0.05 vs. LLD-Li + ASA. Abbreviations: ASA—acetylsalicylic acid, LLD—low-low dose, Li—lithium, STD—standard dose.

No significant difference in creatinine blood levels was found between aspirin (only)-treated rats and control rats on treatment days 21 (Figure 3a, *p* = 0.808) and 42 (Figure 3b, *p* = 0.711). On the other hand, creatinine levels were significantly increased in the LLD-Li and the STD-Li groups both on day 21 and 42 (*p* = 0.0002, *p* < 0.0001, respectively). In comparison to the LLD-Li group, creatinine levels in the LLD-Li + aspirin group were not significantly higher than those in the control group (Figure 3a,b). Once again, these findings support our hypothesis that chronic co-administration of low-dose aspirin and Li does not aggravate the renal side effects of Li.

#### 3.4.2. Cystatin C

**Figure 4:**
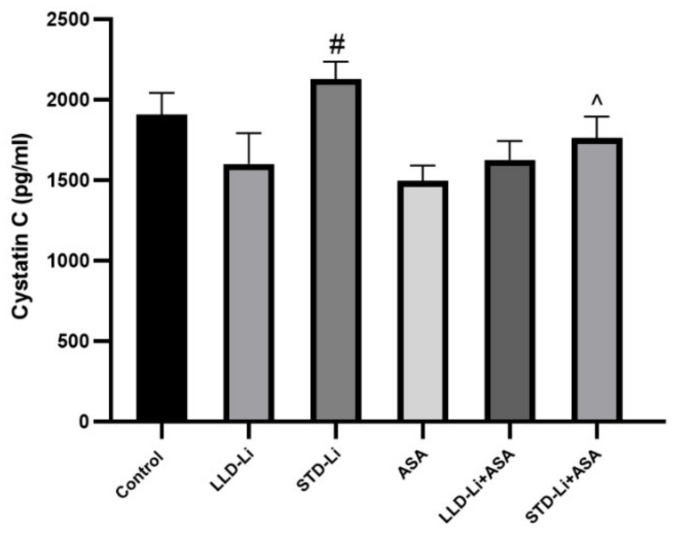
Plasma cystatin C levels in aspirin + Li-treated rats. Rats were fed regular food (control) or lithium-containing food [LLD-Li or STD-Li] for 42 days. Low-dose aspirin (1 mg/kg, ip) was given alone or together with Li. On day 42, rats were euthanized, blood collected, plasma separated, and cystatin C levels determined by ELISA as described in Materials and Methods. Presented are cystatin C levels adjusted to rat’s body weight. Results are the means ±SEM of a single representative experiment out of two demonstrating a similar pattern with 8 rats per group in the depicted experiment. Two-way ANOVA: ASA effect, F_1,40_ = 5.801, *p* = 0.0207; Li effect, F_2,40_ = 3.616, *p* = 0.036; aspirin x Li interaction: F_2,40_ = 1.679, *p* = 0.1995. Post hoc Fisher’s LSD test: Control vs. LLD-Li, *p* = 0.0116; Control vs. STD-Li, *p* = 0.217; Control vs. LLD-Li + ASA, *p* = 0.0118; Control vs. STD-Li + ASA, *p* = 0.413; STD-Li vs. LLD-Li, *p* = 0.008; STD-Li vs. STD-Li + ASA, *p* = 0.0436. Asterisks and symbols denote the following: #—*p* < 0.05 vs. LLD-Li, ^—*p* < 0.05 vs. STD-Li. Abbreviations: ASA—acetylsalicylic acid, LLD—low-low dose, Li—lithium, STD—standard dose.

Chronic treatment with LLD-Li and STD-Li did not significantly increase cystatin C levels, as compared to the control group (*p* = 0.115 and *p* = 0.217, respectively, Figure 4). Cystatin C levels in the STD-Li group were significantly higher as compared to the LLD-Li group (*p* = 0.008, Figure 4). Importantly, cystatin C levels in the aspirin + STD-Li group were significantly lower (*p* < 0.04) than in the STD-Li group (Figure 4), indicating that add-on of low-dose aspirin to Li may reduce the nephrotoxic effects of STD-Li.

#### 3.4.3. Nephrin and Podocin

**Figure 5:**
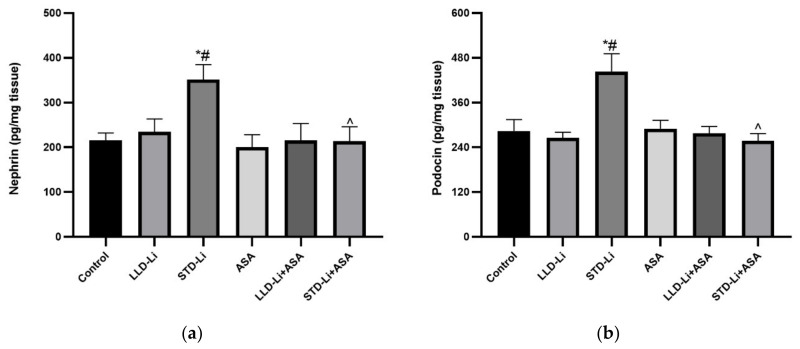
Nephrin and podocin levels in aspirin + Li-treated rats. Rats were fed regular food (control) or lithium-containing food [LLD-Li or STD-Li] for 42 days. Low-dose aspirin (1 mg/kg, ip) was given alone or together with lithium. On day 42 the rats were sacrificed, kidneys excised and homogenized, and nephrin (**a**) and podocin (**b**) levels determined by ELISA as described in Materials and Methods. Results are the means ± SEM of a single representative experiment out of two demonstrating a similar pattern with 8 rats per group in the depicted experiment. * *p* < 0.05 vs. Control, # *p* < 0.05 vs. LLD-Li, ^ *p* < 0.05 vs. STD-Li. (**a**) Nephrin—two-way ANOVA: ASA effect: F_1,30_ = 5.4, *p* = 0.027; Li effect: F_2,30_ = 3.35, *p* < 0.05; Interaction: F_2,30_ = 2.62, *p* = 0.089. Post hoc LSD: Control vs. STD Li, *p* ≤ 0.01; LLD Li vs. STD-Li, *p* ≤ 0.01; STD-Li + ASA vs. STD-Li, *p* = 0.003. (**b**) Podocin—two-way ANOVA: ASA effect: F_1,30_ = 6.018, *p* = 0.02; Li effect: F_2,30_ = 4.449, *p* = 0.02; Interaction: F_2,30_ = 8.121, *p* = 0.0015. Post hoc LSD: Control vs. STD Li, *p* ≤ 0.0004; LLD-Li + vs. STD-Li, *p* ≤ 0.0004; STD Li + ASA vs. STD Li *p* < 0.0001. Abbreviations: ASA—acetylsalicylic acid, LLD—low-low dose, Li—lithium, STD—standard dose.

LLD-Li and aspirin each by themselves did not alter nephrin and podocin levels as compared to control but STD-Li treatment resulted in a significant increase in nephrin and podocin levels in kidney homogenates (*p* < 0.01 and *p* < 0.004, respectively). Importantly, aspirin add-on to STD-Li notably prevented the increase in nephrin and podocin levels (Figure 5a,b, respectively).

#### 3.4.4. Kidney Weight

To exclude renal fibrosis (as seldom reported following long-term Li treatment [39,44,118]), we examined the effect of Li treatment on kidney weight. Kidney weight did not significantly differ between control, Li-treated and aspirin-treated rats following 42 days of treatment: Control = 1.32 ± 0.06 (mean ± SEM, g), LLD-Li = 1.21 ± 0.03, STD-Li = 1.18 ± 0.03, aspirin = 1.36 ± 0.05; *t*-test *p* > 0.05 control vs. other groups.

#### 3.4.5. Renal Histopathology

**Figure 6:**
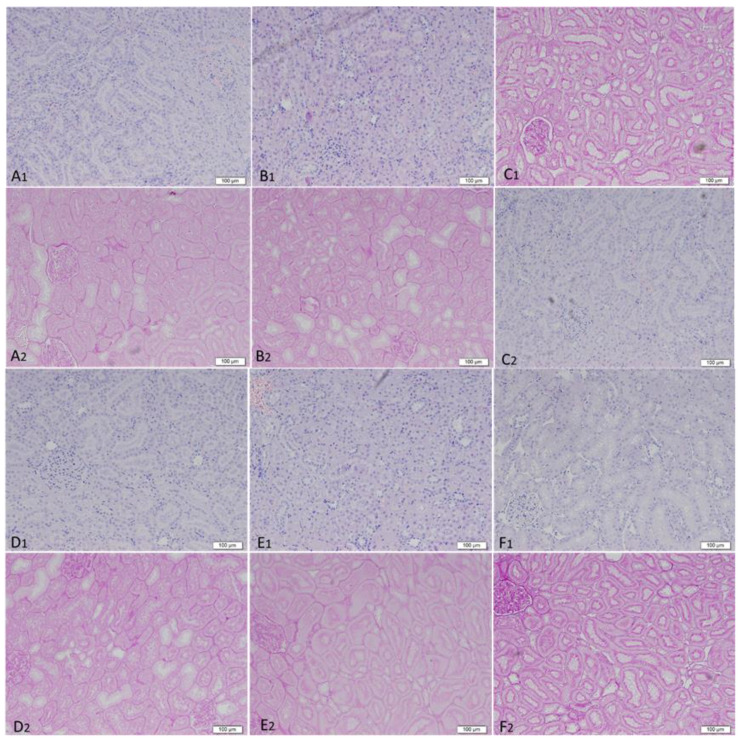
Kidney histopathology in aspirin + Li-treated rats. Rats were fed regular food (control) or lithium-containing food [LLD-Li or STD-Li] for 42 days. Low-dose aspirin (1 mg/kg, ip) was given alone or together with lithium. On day 42, rats were euthanized, kidneys excised, and longitudinally sectioned in half. Then, 2 μm sections were cut, stained with hematoxylin and eosin, and with Periodic acid–Schiff (PAS) and examined as described in Materials and Methods. Hematoxylin and eosin (**A1**–**F1**) and PAS stain (**A2**–**F2**) × 100 magnifications. Six samples from each group were randomly chosen for assessment. A1,2—Control, B1,2—LLD-Li, C1,2—STD-Li, D1,2—Aspirin, E1,2—LLD-Li + aspirin, F1,2—STD-Li + aspirin. Abbreviations: ASA—acetylsalicylic acid, LLD—low-low dose, Li—lithium, STD—standard dose. No discernible differences were found among the groups.

Kidney specimens of the various treatment groups were assessed for histopathological changes, focusing on STD-Li-treated animals (in comparison to control). No tubular atrophy, interstitial fibrosis, tubular cysts or dilatations, or glomerular changes were detected in both Li-treated groups (LLD-Li and STD-Li) as compared to control (Figure 6). Similarly, aspirin (alone or together with Li) did not induce pathological changes in kidney structure.

### 3.5. Effects of Chronic Aspirin plus Li Treatment on Determinants of Gastric Mucosal Integrity

#### 3.5.1. Macroscopic Examination of Stomachs

Stomachs were examined macroscopically for possible gastric side effects including ulceration and bleeding following chronic treatment with aspirin and/or Li. No gastric ulcers/erosions or active bleeding were detected in either of the treatment groups (Table 1). The chi-square test revealed no significant differences between the groups in the rate of mild gastritis (*p* = 0.1049). Few cases of severe gastritis were detected in the STD-Li group (one), aspirin + STD-Li group (one), and in the aspirin-only group (two).

Rats were fed regular food (control) or lithium-containing food [LLD-Li or STD-Li] for 42 days. Low-dose aspirin (1 mg/kg, ip) was given alone or together with lithium. On day 42 rats were sacrificed, stomachs excised, and macroscopic presence of gastric erosions/ulcers was examined by a gastroenterologist as described in Materials and Methods. N = 23–27 rats (stomachs) per group. The table presents the cumulative results of two independent experiments demonstrating a similar pattern. Abbreviations: LLD, low-low dose (Li); STD, standard dose (Li).

#### 3.5.2. Gastric Mucosal PGE2 Levels

**Figure 7:**
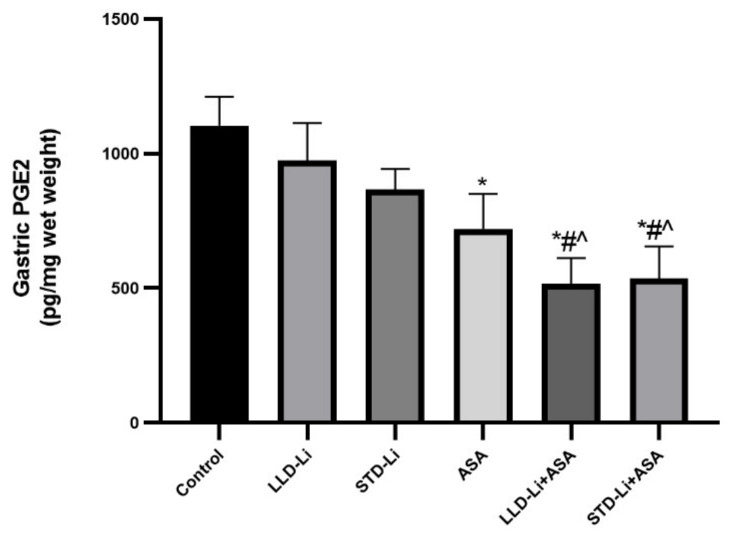
Gastric mucosal PGE2 levels in aspirin + Li-treated rats. Rats were fed regular food (control) or lithium-containing food [LLD-Li or STD-Li] for 42 days. Low-dose ASA (1 mg/kg, ip) was administered alone or together with Li. On day 42 rats were euthanized, stomachs excised, 100 mg of the gastric tissue homogenized and mucosal PGE2 levels determined by ELISA as described in Materials and Methods. Results are the means ± SEM of 18 rats per group. The figure summarizes the combined results of two independent experiments demonstrating a similar pattern. Two-way ANOVA: ASA effect, F1,101 = 7.83, *p* < 0.0001; Li effect, F_2,101_ = 1.926, *p* = 0.151; aspirin x Li interaction: F2,30 = 0.1592, *p* = 0.8531. Post hoc Fisher’s LSD test: Control vs. LLD-Li, *p* = 0.419; Control vs. STD-Li, *p* = 0.14; Control vs. ASA, *p* = 0.018; Control vs. LLD-Li + ASA, *p* = 0.0004; Control vs. STD-Li + ASA, *p* = 0.0007; ASA vs. LLD-Li + ASA, *p* = 0.2037; ASA vs. STD-Li + ASA, *p* = 0.2576; LLD-Li vs. LLD-Li + ASA, *p* = 0.005; STD-Li vs. STD-Li + ASA, *p* = 0.044. Asterisks and symbols denote the following: *—*p* < 0.05 vs. Control, #—*p* < 0.05 vs. LLD-Li, ^—*p* < 0.05 vs. STD-Li. Abbreviations: ASA—acetylsalicylic acid, LLD—low-low dose, Li—lithium, STD—standard dose.

PGE2 acts as a protective mediator in the stomach by inhibiting the secretion of hydrochloric acid and enhancing the secretion of bicarbonate and mucus, all of which decrease the risk of ulceration and bleeding. Mucosal PGE2 was determined to elucidate whether aspirin and/or Li treatment induce a decrease in PGE2 levels and, thus, increase the risk for gastric ulceration and bleeding. As seen in Figure 7, treatment with aspirin alone led to a significant decrease in mucosal PGE2 levels as compared to control (*p* = 0.0018), whereas LLD-Li and STD-Li did not cause a significant decrease. Aspirin add-on to each of the Li groups (LLD-Li and STD-Li) did not further significantly reduce PGE2 levels beyond those of the aspirin (only) group, suggesting that the prominent decrease in PGE2 levels in these groups derived mainly from the effect of aspirin.

#### 3.5.3. Gastrointestinal Bleeding

**Figure 8:**
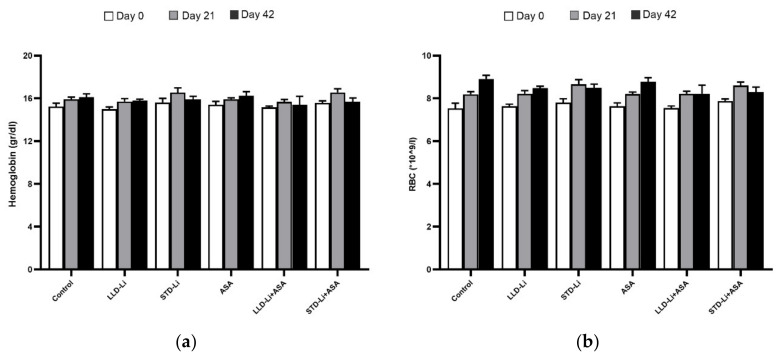
Blood hemoglobin and red blood cell levels in aspirin + Li-treated rats. Rats were fed regular food (control) or lithium-containing food [LLD-Li or STD-Li] for 42 days. Low-dose ASA (1 mg/kg, ip) was administered alone or together with Li. On the indicated days, blood was collected, and hemoglobin levels and red blood cell count were measured as described in Materials and Methods. Results are the means ± SEM of a single representative experiment out of two demonstrating a similar pattern with 6–8 rats per group in the depicted experiment. (**a**) Hemoglobin—One-way ANOVA: Day 0—*p* = 0.547; day 21—*p* = 0.07; day 42—*p* = 0.29. (**b**) RBC—One-way ANOVA: Day 0—*p* = 0.547; day 21—*p* = 0.07; day 42—*p* = 0.294. Abbreviations: ASA—acetylsalicylic acid, LLD—low-low dose, Li—lithium, STD—standard dose.

Aspirin and/or Li treatment was not associated with a significant change in red blood cell count and hemoglobin levels as compared to the control (Figure 8).

#### 3.5.4. Plasma TXA2 Levels

**Figure 9:**
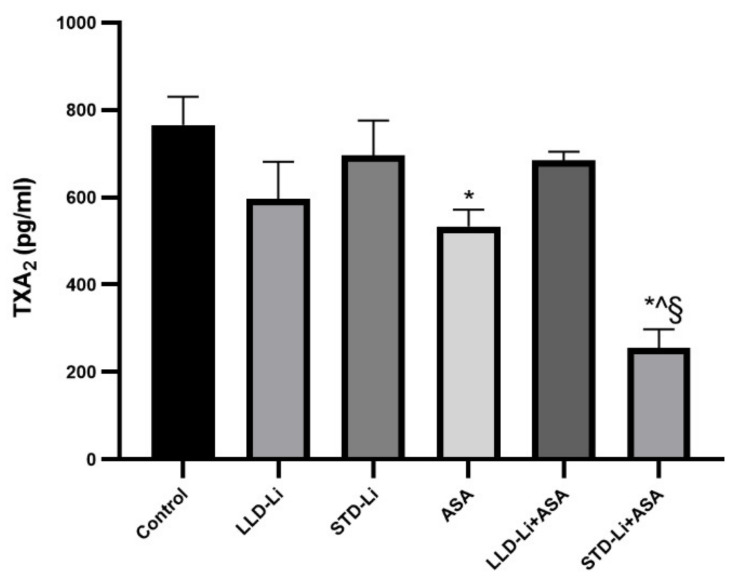
Plasma TXA2 in aspirin + lithium-treated rats. Rats were fed regular food (control) or lithium-containing food [LLD-Li or STD-Li] for 42 days. Low-dose ASA (1 mg/kg, ip) was administered alone or together with Li. On day 42 rats were sacrificed, blood collected, serum separated, and TXA2 levels determined by ELISA as described in Materials and Methods. Results are the means ± SEM of a single representative experiment out of two demonstrating a similar pattern with 6–8 rats per group in the depicted experiment. Two-way ANOVA: ASA effect, F_1,40_ = 4.204, *p* = 0.046; Li effect, F_2,40_ = 5.071, *p* = 0.01; aspirin x Li interaction: F_2,40_ = 3.312, *p* = 0.046. Post hoc Fisher’s LSD test: Control vs. LLD-Li, *p* = 0.107; Control vs. STD-Li, *p* = 0.077; Control vs. ASA, *p* = 0.048; Control vs. LLD-Li + ASA, *p* = 0.426; STD-Li vs. STD-Li + ASA, *p* = 0.0135; ASA vs. STD-Li + ASA, *p* = 0.0233; STD-Li vs. STD-Li + ASA, *p* = 0.044. Asterisks and symbols denote the following: *—*p* < 0.05 vs. Control, ^—*p* < 0.05 vs. STD-Li, §—*p* < 0.05 vs. ASA. Abbreviations: ASA—acetylsalicylic acid, LLD—low-low dose, Li—lithium, STD—standard dose.

TXA2 is a metabolite of arachidonic acid that stimulates platelet aggregation and causes vasoconstriction. Inhibition of TXA2 synthesis by aspirin is the major mechanism underlying its antiplatelet effect. We, therefore, tested the effects of aspirin/Li treatment on plasma TXA2 levels to determine whether the experimental conditions used lead to a reduction in TXA2 levels, consistent with the therapeutic antiplatelet effect of aspirin. As seen in Figure 9, aspirin by itself indeed significantly decreased plasma TXA2 levels. However, co-administration of aspirin together with LLD-Li did not result in a significant reduction in TXA2 levels but aspirin and STD-Li co-administration did significantly augment the effect of aspirin. Namely, aspirin + STD-Li treatment resulted in significantly lower TXA2 levels than treatment with aspirin alone, suggestive of a possible synergistic effect of the combined treatment. Nonetheless, these results seem clinically insignificant because there were no significant differences in signs of bleeding between aspirin or/and lithium-treated rats, as compared to the control.

### 3.6. Behavioral Effects of Chronic Low-Dose Aspirin Plus Li Treatment—Proof of Concept Experiments

These experiments were conducted to test the hypothesis that the administration of low-dose aspirin will enhance the therapeutic effects of low-dose Li. We tested the effects of the combinatory treatment on several behavioral phenotypes characteristic of mood disorders, using accepted behavioral models.

#### 3.6.1. Anxiety-Like Behaviors

**Figure 10:**
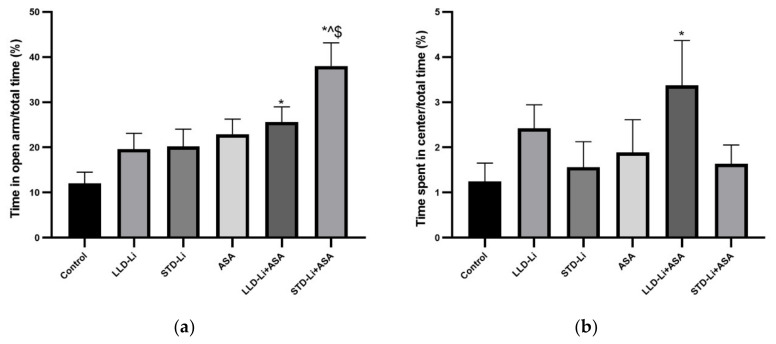
Anxiety-like behavioral facets in aspirin + Li-treated rats. Rats were fed regular food (control) or Li-containing food [LLD-Li or STD-Li] for 42 days. Low-dose ASA (1 mg/kg, ip) was administered alone or together with Li. (**a**) On treatment day 14, rats were subjected to an EPMT for five minutes. (**b**) On day 35, rats were placed in an open field arena for 20 min and their locomotor activity was monitored. In both tests, rats’ behavior was videotaped and subsequently analyzed by a video-tracking system as described in Materials and Methods. (**a**) Results are the means ± SEM of a single representative experiment out of two demonstrating a similar pattern with 9–12 rats per group in the depicted experiment. Two-way ANOVA: ASA effect, F_1,60_ = 13.38, *p* = 0.0005; Li effect, F_2,60_ = 406, *p* = 0.016; aspirin x Li interaction: F_2,60_ = 1.295, *p* = 0.2813. Post hoc Fisher’s LSD test: Control vs. LLD-Li, *p* = 0.1797; Control vs. STD-Li, *p* = 0.1491; Control vs. ASA, *p* = 0.0739; Control vs. LLD-Li + ASA, *p* = 0.0182; Control vs. STD-Li + ASA, *p* < 0.0001; STD-Li vs. STD-Li + ASA, *p* = 0.0011; LLD-Li + ASA vs. STD-Li + ASA, *p* = 0.0205. Asterisks and symbols denote the following: *—*p* < 0.05 vs. control, ^—*p* < 0.05 vs. STD-Li, $—*p* < 0.05 vs. LLD-Li + ASA. (**b**) Results are means ± SEM of a single representative experiment out of two demonstrating a similar pattern with 9–12 rats per group in the depicted experiment. Two-way ANOVA: ASA effect, F_1,60_ = 1.069, *p* = 0.3052; One-tailed Li effect, F_2,60_ = 2.804, *p* = 0.0329; aspirin x Li interaction: F_2,60_ = 0.2523, *p* = 0.7778. Post hoc Fisher’s LSD test: Control vs. LLD-Li, *p* = 0.2228; Control vs. STD-Li, *p* = 0.7405; Control vs. ASA, *p* = 0.5316; Control vs. LLD-Li + ASA, *p* = 0.0296; Control vs. STD-Li + ASA, *p* = 0.6827. Asterisk denotes the following: *—*p* < 0.05 vs. Control. Abbreviations: ASA—acetylsalicylic acid, LLD—low-low dose, Li—lithium, STD—standard dose.

Anxiety-like and risk-taking (opposite of anxiety) behaviors were assessed by measuring (1) the time spent in the closed vs. open arms in the EPMT (Figure 10a), (2) the time spent in the peripheral vs. central zone in the OFT (Figure 10b). Fourteen days of treatment with LLD-Li and STD-Li did not significantly increase the time spent in the open arms (Figure 10a). In contrast, co-administration of aspirin together with Li significantly increased the time spent in the open arms, suggestive of an anxiolytic-like effect of the combined treatments (Figure 10a). In the OFT, treatment with LLD-Li, or STD-Li, or aspirin, each by itself, did not significantly increase the time spent in the center of the arena, as compared to control (Figure 10b). In contrast, treatment with aspirin add-on to LLD-Li, as compared to control, significantly increased the time spent in the center of the arena, suggestive of an anxiolytic-like effect of the combined treatment. Unexpectedly, aspirin + STD-Li treatment did not alter the measures of this test (Figure 10b). Together with the results of the EPMT (Figure 10a), these findings support the notion that low-dose aspirin + low-dose Li induce an anxiolytic-like effect.

#### 3.6.2. Depressive-Like Behavior

**Figure 11:**
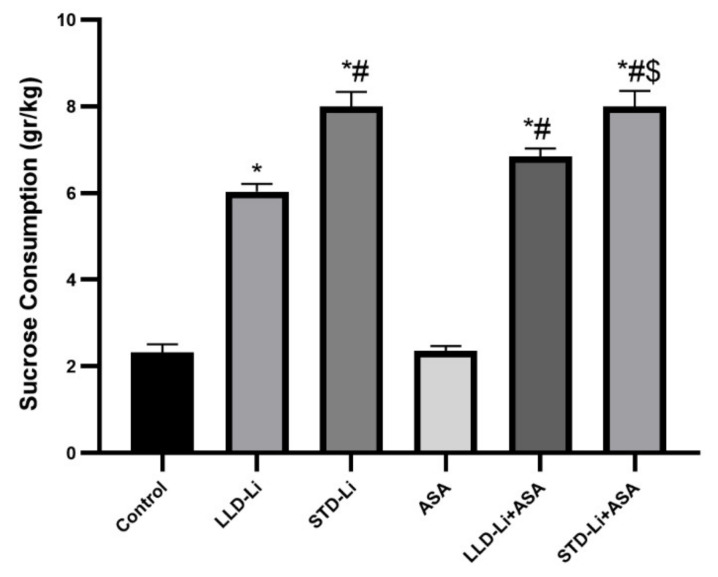
Rats were fed regular food (control) or lithium-containing food [LLD-Li or STD-Li]. On day 21 of the treatment rats were offered two bottles, one containing sucrose solution and one—regular drinking water as described in Materials and Methods. Sucrose consumption in each cage was calculated according to the body weight of the rats (three) in the cage. Results are the means ± SEM of a single representative experiment out of two demonstrating a similar pattern with 9–12 rats per group in the depicted experiment. Two-way ANOVA: ASA effect, F_1,48_ = 1.711, *p* = 0.1738; Li effect, F_2,48_ = 278.7, *p* < 0.0001; aspirin x Li interaction, F_2,48_ = 1.711, *p* = 0.1915. Post hoc Fisher’s LSD test: Control vs. LLD-Li, *p* < 0.0001; Control vs. STD-Li, *p* < 0.0001; Control vs. ASA, *p* = 0.9362; Control vs. LLD-Li + ASA, *p* < 0.0001; Control vs. STD-Li + ASA, *p* < 0.0001; LLD-Li vs. STD-Li; LLD-Li vs. LLD-Li + ASA, *p* = 0.0254; STD-Li vs. STD-Li + ASA, *p* = 0.997; LLD-Li + ASA vs. STD-Li +ASA, *p* = 0.0019. Asterisks and symbols denote the following: *—*p* < 0.05 vs. Control, #—*p* < 0.05 vs. LLD-Li, $—*p* <, 0.05 vs. LLD-Li + ASA. Abbreviations: ASA—acetylsalicylic acid, LLD—low-low dose, Li—lithium, STD—standard dose.

Anhedonia is one of the prominent features of depression. Hedonic-like behavior was assessed using the SCT. At baseline, there was no difference in sucrose solution consumption between the groups (data not shown). Figure 11 demonstrates that on day 21, sucrose consumption was significantly higher in the two Li-treated groups as compared to the control group (*p* < 0.0001), and significantly higher in the STD-Li than in the LLD-Li group (*p* < 0.0001). Aspirin by itself did not affect sucrose consumption but its co-administration with LLD-Li significantly augmented the hedonic-like behavior of LLD-Li- treated rats (*p* = 0.0254).

## 4. Discussion

A two-fold rationale inspired the present study of add-on low-dose aspirin to Li treatment. First, among its beneficial characteristics Li has been shown to exert anti-neuro-inflammatory/anti-inflammatory effects [70,73]. Given that low-dose aspirin has also been reported to exert anti-inflammatory effects [119,120] along with the well-established involvement of inflammation in the pathophysiology of BD [45,46,47,48,49], we hypothesized that co-administration of low-dose aspirin and Li could enable reduction in the required Li dose to gain mood stabilization. Second, prior to the above, given the devastating renal side effects of Li treatment in BD—NDI, and even CKD following long-term use, we first probed the safety of chronic administration of aspirin plus Li. In this context, it is worth noting that people with CKD face between two to five times higher risk than the general population of enduring cardiovascular events and stroke [121,122]. In an ongoing study funded by the National Institute of Health Research and the British Heart Foundation, a group of scientists from the University of Southampton are currently testing the hypothesis that taking a low-dose aspirin tablet once daily reduces the risk of acute cardiovascular events and strokes in people with CKD who do not have pre-existing cardiovascular disease (Trial Registration: NCT03796156). To the best of our knowledge, the present study is the first to address in a systematic manner in rodents both the safety and the efficacy of low-dose aspirin added on to Li treatment.

Here we show that co-treatment of low-dose aspirin and low-dose Li is as safe as low-dose Li only, and that co-treatment with low-dose aspirin and STD-Li is probably safer than STD-Li by itself, as it attenuated some aspects of STD-Li-induced nephrotoxicity. Namely, co-treatment with low-dose aspirin and Li did not alter plasma Li levels in all regimens of Li administration, indicating that the combined treatment is safe and does not increase the risk for lithium intoxication. In the 1980s, of the previous century, some inconsistent reports of murine and human studies [123,124,125] argued whether or not co-administration of aspirin and Li affects serum Li levels. The use of a German drug combination, an over-the-counter analgesic titled ‘Togal,’ containing the active ingredients of aspirin, Li-citrate, and quinine, sold from the early 20th century till 2011, was also reported [126,127], but we were not able to locate reports of its mood-related or systemic side effects. Later on, in the last years of the 20th century, the debate over whether NSAIDs, in general, and aspirin, in particular, interact with Li in a toxic-synergistic manner, was raised again [128,129]. More recent studies suggested that concomitant use of low-dose aspirin with Li is safe and does not increase Li toxicity [130,131]. For instance, in a randomized, double-blind controlled study of patients with BD maintained on Li and aspirin (240 mg/day) vs. Li and placebo, plasma Li concentrations in the aspirin plus Li group did not differ significantly from those of the Li plus placebo group at the end of the study [131]. Beyond providing a clear-cut answer to the above inconsistency, our finding is important in view of the fact that COX inhibitors may decrease PG synthesis and attenuate renal blood flow and glomerular filtration rate, thus leading to a reduction in lithium clearance and elevation in plasma concentrations of the drug [31,106,123,130,131,132].

Our findings that chronic STD-Li treatment resulted in significant polyuria and polydipsia (characteristic features of NDI) (Figure 2), and in elevated blood levels of creatinine and cystatin C (markers of abnormal renal function; Figure 3 and Figure 4) are in agreement with the strong evidence that long-term Li treatment is associated with renal function abnormalities [30,31,33]. Low-dose aspirin significantly decreased the STD-Li-induced increase in water consumption and urinary output at 21 and 42 days of treatment as compared to STD-Li only (Figure 2). If confirmed in humans, this finding suggests that co-administration of low-dose aspirin together with Li may reduce the polydipsia and polyuria in Li-treated BD patients.

The progressive increase (0.85 mmol/L on day 21 and 0.95 mmol/L on day 42) in blood creatinine levels in the STD-Li group is compatible with the reports of the correlation between treatment duration and CKD [31,35]. Chronic co-administration of low-dose aspirin and Li did not aggravate the renal side effects of Li. Serum creatinine levels in the aspirin plus STD-Li treated group were similar to those in the STD-Li (only) group at 21 and 42 days of treatment (Figure 3). Importantly, co-treatment with low-dose aspirin prevented a significant elevation in creatinine levels in LLD-Li-treated rats (Figure 3), suggestive of a protective effect of aspirin treatment.

Cystatin C has been suggested as a biomarker that may help in detecting early impairment in glomerular filtration [109,110]. However, it is important to bear in mind that many factors affect the blood levels of cystatin C [133] and may bias the results of a given intervention. Interestingly, a study in BD patients revealed that long-term Li treatment did not alter serum cystatin C levels [134]. In the present study, STD-Li treatment was associated with a significant increase in plasma cystatin C levels (Figure 4), attenuated by co-treatment with low-dose aspirin. This is consistent with the protective effect of aspirin against Li-induced nephrotoxicity.

Nephrin and podocin play a crucial role in podocyte function [111] and regulation of glomerular function [112,113]. Studies that tested the effects of Li on nephrin and podocin expression revealed inconsistent results. An in vivo study in mice showed that a single injection of Li caused a decrease in nephrin expression in kidney lysate [135]. On the other hand, a study in obese mice demonstrated that chronic Li treatment which resulted in plasma concentrations between ~0.25–0.5 mM did not alter nephrin and podocin mRNA levels [136]. In the present study, chronic STD-Li treatment resulted in a significant increase in nephrin and podocin levels in kidney homogenates (Figure 5). Currently, the mechanism underlying this observation is unknown and it is also unclear whether it represents a positive or negative pathophysiological outcome. It is also unknown whether the increase is a time-dependent phenomenon and whether a longer treatment duration would diminish or intensify its manifestation. Since nephrin and podocin are essential proteins for normal glomerular function [111,112,113], an increase in their levels, to a certain extent, may theoretically represent a positive outcome of Li treatment. Consistent with this assumption, and in contrast with the prevailing view, several experimental studies in animals have shown that Li may exert positive effects on renal function through inhibition of the enzyme glycogen synthase kinase (GSK)-3β [137,138,139,140,141]. For example, in a mouse model of doxorubicin-induced nephropathy, a single dose of Li improved glomerular function and attenuated the proteinuria and glomerulosclerosis associated with this condition [140]. Similarly, pretreatment with Li significantly reduced proteinuria and improved glomerular function in a mouse model of doxorubicin- or lipopolysaccharide-induced podocyte injury in mice [141]. Consistent with these findings, selective pharmacological or genetic inhibition of GSK-3β was shown to improve renal function in various models of kidney disorders in animals [142,143,144]. Support for these experimental data comes from clinical evidence indicating that chronic Li treatment leads to a slight (if any) decrease in glomerular function and a negligible incidence of CKD that are of questionable clinical significance [30,31,41,42,145]. Interestingly, in the present study, low-dose aspirin co-treatment totally reversed the effects of STD-Li on nephrin and podocin levels (Figure 5).

It has been suggested that administration of low to medium Li doses may reduce the incidence of CKD [31,41,42,43,146]. In our study, LLD-Li administration led to a less severe impairment in renal function as compared to STD-Li treatment. Coadministration of aspirin with LLD-Li did not aggravate this effect. Furthermore, while creatinine levels in the LLD-Li-treated animals were significantly increased, they did not differ significantly from those in control animals throughout the whole treatment duration in the aspirin plus LLD-Li group, suggestive of a protective effect.

Long-term administration of Li has been reported to induce chronic tubulointerstitial damage in humans [35,39] and rats [118,147,148]. These studies reported renal biopsies presenting tubular atrophy, microcystic changes of the distal tubule, cortical and medullary tubular cysts, and interstitial fibrosis [35,39,118,147,148]. The degree of interstitial fibrosis in the biopsy was related to the duration of treatment and Li’s cumulative dose modeling. However, contradicting findings were reported as well, namely, no difference between Li-treated patients and those who did not receive the drug [149]. Furthermore, it is difficult to determine whether or not there is an association between long-term Li treatment and tubulo-interstitial damage as very few patients on Li therapy undergo a renal biopsy and only a relatively small percentage of patients present with advanced renal disease [31,35]. In an attempt to replicate the slowly progressive chronic interstitial fibrosis, Walker et al. [148] developed an animal model of Li-induced chronic interstitial fibrosis. In this model, progressive development of renal fibrosis was detected in rats treated with a therapeutically relevant dose (0.8–1.3 mEq/L) of Li over 6 months. In the present study, renal histology did not demonstrate the development of fibrosis following 42 days of Li treatment. Importantly, no tubulo-interstitial damage was detected in either aspirin (only) or aspirin and Li-treated rats. Taking into account that long-term Li treatment duration is a risk factor for CKD [30,31,32,33,34,35,36,37,38,39], one possible explanation for the discrepancy between our results and those of Walker et al. [148] could be the longer treatment duration in the Walker study (6 weeks vs. 24 weeks, respectively).

The parameter of gastric mucosal PGE2 levels was used to evaluate whether aspirin and/or Li treatment results in an increased risk for gastric ulceration and bleeding. Compatible with the general knowledge that most patients who take aspirin develop acute mucosal lesions while Li treatment is not associated with digestive tract maladies, chronic administration of aspirin indeed led to a significant decrease in gastric mucosal PGE2 levels as compared to control (Figure 7), whereas the decrease induced by Li-only treatment did not reach statistical significance. In any event, the decrease in gastric PGE2 levels did not lead to prominent mucosal damage, as no ulcers were detected in any treatment group after 42 days of treatment (Table 1). Furthermore, all treatment groups demonstrated stable red blood cell count and hemoglobin levels throughout the study, and chronic low-dose aspirin plus Li treatment was not associated with a significant increase in gastrointestinal bleeding.

In humans, chronic low-dose aspirin is mostly prescribed for the prevention of adverse cardiovascular events, due to its potent antiplatelet effect [85,86,87] obtained mainly through inhibition of platelet TXA2 synthesis [25,84]. To examine whether our treatment protocol leads to a desired therapeutic effect of low-dose aspirin, we determined the effect of the various treatment regimens on plasma TXA2 levels (Figure 9). As predicted, aspirin treatment significantly decreased plasma TXA2 levels as compared to control (Figure 9), confirming its antiplatelet effect under the experimental conditions of the study. Furthermore, aspirin and STD-Li co-administration significantly augmented the effect of aspirin. Therefore, our results suggest a potential for the combined low-dose aspirin plus low-dose Li to reduce the risk of major adverse cardiovascular events in BD patients.

The behavioral experiments of the present study aimed to test the hypothesis that the addition of low-dose aspirin to low-dose Li would preserve the therapeutic efficacy of Li. In humans, the therapeutic window of Li for maintenance treatment of BD patients is between 0.6 and 1.2 mEq/liter [25,26], while lower concentrations (0.4–0.8 mEq/liter) are recommended for geriatric patients [150]. In the present study, chronic LLD-Li (plasma levels ~0.4 mEq/liter) treatment significantly increased sucrose consumption (Figure 11), interpretable as an antidepressant-like effect. As expected, STD-Li treatment also caused a significant increase in sucrose consumption (Figure 11). Importantly, aspirin add-on to LLD-Li significantly augmented the antidepressant-like effect of LLD-Li. As for the anxiety-like behavior, LLD-Li led to a non-significant increase in the time spent in the open arms of the elevated plus-maze and in the central zone of the open field (Figure 10a,b, respectively), but add-on of low-dose aspirin to LLD-Li resulted in a significant increase in the time spent in the open arms of the elevated plus-maze and in the central zone of the open field as compared with the LLD-Li only group, suggestive of an anxiolytic-like effect of the combined treatment. These findings support the use of low-dose aspirin with low-dose Li to augment positive Li-induced mood-modulating effects while minimizing the drug’s toxicity. Surprisingly, STD-Li treatment was not associated with a significant effect on these anxiety-related tests, neither by itself nor in combination with low-dose aspirin. This finding corroborates with a study in adolescent rats which showed that chronic Li treatment lacked an anxiolytic-like effect and even induced anxiety-like behavior [151].

The biological basis of Li-induced NDI is very complex and seems to involve a variety of mechanisms (for review see [152]). The severity and reversibility of Li-induced NDI differ among patients and seem to be affected by the duration of Li treatment and the stage of tubulointerstitial damage [31]. Initially, Li treatment may induce only functional tubulointerstitial damage which may be reversible upon Li cessation. However, if long-term Li treatment causes irreversible morphological tubulointerstitial damage (fibrosis), discontinuation of the drug most probably will not resolve the problem [31]. In the present study, we found that low-dose aspirin significantly mitigated the STD-Li-induced increase in urinary output (Figure 2). The mechanism underlying this protective effect is not fully understood. In the kidneys, Li enters the principal cells of the collecting duct through epithelial sodium channels in the luminal membrane [152,153,154]. It then accumulates in these cells and interferes with the ability of the antidiuretic hormone to increase water permeability. Li inhibits GSK-3β leading to increased expression of COX-2 and over-production of PGE2, which suppresses the antidiuretic effect of vasopressin resulting in increased urination [152]. PGE2 acts on principal cells to induce lysosomal degradation of AQP2 water channels and a decline in urine concentrating ability. It is possible that low-dose aspirin attenuates the polyuria-inducing effect of Li by counteracting its effect on the GSK-3β-COX-2-PGE2-vasopressin cascade by inhibiting PGE2 production and restoring the antidiuretic effect of vasopressin. Of note, GSK-3β activity is crucial for optimal renal physiology, in general [152] and essential for podocyte function in particular [155]. Aberrant kidney GSK-3 β activity leads to severe albuminuria and renal failure [155]. Inhibition of GSK-3β has been associated with potent antiapoptotic effects and accumulation of β-catenin (among other down-stream targets) [156,157,158]. Alterations in the Wnt/β-catenin signaling pathway are associated with various pathophysiological features including cancer promotion [159] and impairment of kidney function [135,136,152,155]. Interestingly, it was found that aspirin dose-dependently inhibits the activity of the Wnt/β-catenin pathway [160,161]. Therefore, it is possible that in addition to other modes by which aspirin reduces Li-induced nephrotoxicity, it also neutralizes the effects of Li on the β-catenin pathway.

The mechanism by which low-dose aspirin add-on therapy to Li may enhance the therapeutic efficacy of Li is yet to be unraveled. It is generally accepted that when aspirin is given at a low dose for platelet inhibition, it exerts weak anti-inflammatory effects because it preferentially inhibits COX-1 [25]. In this regard, Fritz et al. [162] demonstrated that inhibition of brain COX-1 (but not COX-2) mitigated inflammation-induced depressive-like behavior in mice through inhibition of PGE2 production/activity in the striatum. In our study, chronic treatment with low-dose aspirin in rats reduced PGE2 levels in the frontal cortex but not in the hypothalamus or hippocampus (data not shown). Thus, the fact that low-dose aspirin preferentially inhibits COX-1 [25,161] does not necessarily mean that it entirely lacks an anti-inflammatory effect. It is also possible that COX-independent anti-inflammatory and non-inflammatory-associated mechanisms contribute to the beneficial behavioral effects of low-dose aspirin. For example, some [163,164] (but not all [165,166]) studies report that, similarly to Li, aspirin confers autophagy-augmenting effects raising the possibility that autophagy enhancement is a common mood-stabilizing mechanism of Li and aspirin.

There are additional potential benefits of adding aspirin to the treatment of BD patients. First, as mentioned above, considering that BD patients have an increased risk of mortality from cardiovascular disease [8,10,14], the antithrombotic effect of aspirin may reduce mortality among these patients. However, it is worth emphasizing that the efficacy and safety of aspirin for primary prevention of adverse cardiovascular events in the general population—subjects without known cardiovascular diseases—remains controversial [167,168,169] and necessitates further research. Second, aspirin was shown to effectively improve Li-related sexual dysfunction in men with stable BD [131]. Third, long-term use of aspirin has been associated with a reduced incidence of various types of cancer [74,170,171,172,173,174,175,176].

## 5. Conclusions

In summary, we demonstrate that co-administration of low-dose aspirin with low-dose Li mitigates typical renal side-effects of standard-dose Li while retaining the beneficial behavioral effects of this enigmatic cation.

## Figures and Tables

**Table 1:** Gastric mucosal integrity in aspirin + Li-treated rats.

Title 1	Control	LLD-Li	STD-Li	Aspirin	LLD-Li + Aspirin	STD-Li + Aspirin
N	23	25	27	24	27	27
Mild gastritis, n (%)	11 (48)	12 (48)	18 (67)	10 (42)	16 (59)	19 (70)
Severe gastritis, n (%)	0	0	1 (3.7)	2 (8.3)	0	1 (3.7)
Gastric bleeding	0	0	0	0	0	0
Gastric ulcer	0	0	0	0	0	0

## Data Availability

The datasets used and analyzed in the study are available from the corresponding author upon reasonable request.
